# Characteristics of Children with Cerebral Palsy and Their Utilization of Services in Saudi Arabia

**DOI:** 10.3390/healthcare11192690

**Published:** 2023-10-07

**Authors:** Maysoun Nimer Saleh, Ahmad Alharbi, Abdulaziz Albalwi, Salem Alatawi, Maaidah Algamdi, Abdulaziz Alshahrani, Basil Al Bakri, Nihad Almasri

**Affiliations:** 1 Department of Physical Therapy, Faculty of Applied Medical Sciences, University of Tabuk, Tabuk 71491, Saudi Arabia; aaalharbi@ut.edu.sa (A.A.); aa-albalwi@ut.edu.sa (A.A.); sfalatawi@ut.edu.sa (S.A.); zezovic1@gmail.com (A.A.); 2 Department of Physiotherapy, School of Rehabilitation Sciences, The University of Jordan, Amman 11942, Jordan; n.almasri@ju.edu.jo; 3 Department of Nursing, Faculty of Applied Medical Sciences, University of Tabuk, Tabuk 71491, Saudi Arabia; ialghamdi@ut.edu.sa; 4 Department of General Surgery, School of Medicine, The University of Jordan, Amman 11942, Jordan; basilbakri@gmail.com

**Keywords:** cerebral palsy (CP), children, Saudi Arabia, service utilization, GMFCS

## Abstract

The recent emergence of research on cerebral palsy (CP) in developing countries aims to improve knowledge on affected children and the utilization of the available services. This study seeks to describe children with CP in Saudi Arabia and service utilization as per Gross Motor Function Classification System (GMFCS) levels and geographic regions. A cross-sectional survey of 227 children with CP (Mean age 6.3, SD 3.9 years) was conducted. Parents reported on children’s demographics, impairments, and service utilization. Half of the children (*n* = 113, 49.8%) had ≥3 impairments with speech, visual and learning impairments being the most frequent. The total number of impairments differed significantly by GMFCS, *F* (4, 218) = 8.87, *p* < 0.001. Most of the children (*n* = 86, 83.4%) used 2–5 services. Moreover, 139 (62.3%) did not attend school, 147 (65.9%) did not receive occupational therapy, and only 32 (14.3%) received speech therapy. More children in GMFCS level I did not receive neurologist services. Profiles of children and services were described by GMFCS and by regions. This was the first study to describe children with CP and service utilization in Saudi Arabia. Although many impairments affected the children, there was low utilization of related services. Data on service utilization and on unmet needs support a comprehensive approach to rehabilitation and the proper service allocation.

## 1. Introduction

Cerebral palsy (CP) is the most common neurodevelopmental disability in childhood, with an estimated birth prevalence in high-income countries of 1.6 per 1000 live births [1], while the estimated prevalence in Arab-speaking countries is 1.8 per 1000 live births [2]. Although CP is mainly a disorder of posture and movement, it encompasses a variety of sensation, cognition, communication, perception, emotional, and behavioral impairments leading to functional limitations and participation restrictions of the affected child [3,4,5]. Therefore, children and their families experience a considerable amount of stress [4,6,7] and are more likely to face restricted access to health services than those without chronic illnesses or disabilities [8]. Indeed, children with disabilities face long waiting times leading to delays in accessing the needed services [9,10].

Guidelines on the provision of quality medical services for children with CP are not yet conclusive [11,12]. Most of the existing guidelines may not reflect the needs of the child and family because of decreased child and parent input [11]. However, providing the best available services for children with CP first requires the exploration of the characteristics of these children and their caring families, as well as identifying the existing services. Services provided for people with CP might vary based on child and family characteristics [13]. In addition, children in different geographic regions of the same country may receive different levels of physical therapy services [12].

The level of gross motor functioning in children with CP was shown to be important in determining the type of services these children receive, as well as influencing parental needs and concerns [14]. In addition, Al Imam et al. [13] reported that decreased use of rehabilitation services was significantly associated with being a female child with lower maternal and parental education, lower family income, hearing impairment, and severe motor disability (GMFCS III–V). Ryan et al. [15] described patterns of visits to rehabilitation and medical professionals among ambulatory (GMFCS I-III) young people (10–19 years old) with CP living in England, and factors associated with service use. They reported that physical therapists were the most visited professionals, followed by dentists, general practitioners, occupational therapy, and orthopedic surgeons. Age, GMFCS level, and speech impairment were identified factors affecting service use.

In Arab-speaking countries, epidemiological data on CP are limited [2,16,17]. In Jordan, Saleh and Almasri [16] described profiles of children with CP and services and they covered a wide range of age groups (1–17 years). They reported that spastic CP (74.1%) was the most common type of CP, with speech and visual impairments reported as the most common associated impairments. In addition, physical therapy (PT) was the most frequent service received (90.4%). Factors related to service use in Jordan were related to family resources, child needs and associated impairments, and satisfaction with services [18]. Mushta et al. [2] conducted a systematic review of research studies on the epidemiology of CP in Arab-speaking countries (32 studies from 7 Arab countries), which showed spastic quadriplegia to be the most common type of CP. In this systematic review, only one study from Iraq reported on services received by children with CP. The aforementioned study by Mushta et al. [2] highlighted knowledge gaps on CP in Arab-speaking countries.

Saudi Arabia is a country with a population of almost 32 million and a median age of 22 years (24.5% of the population are children, 61.2% are males [19]). However, a detailed description of children with CP including impairments and functional limitations is lacking. Most of the studies on CP in Saudi Arabia were restricted to a certain region or city [17,20]; or they were mainly concerned with prevalence [21]. The characteristics of children with CP and the services available to them are areas yet to be explored. This information is expected to guide policymakers in planning services for children with CP and their families. Therefore, the aims of this study were (1) to investigate the characteristics of children with CP (e.g., demographics, associated impairments, and motor function levels) and their families (e.g., age of parents, education, family income, etc.) in Saudi Arabia; (2) to describe their service (education, medical, and rehabilitation) utilization including frequency, intensity, and insurance coverage; and (3) to examine how the medical and rehabilitation services used differed by Gross Motor Function Classification System (GMFCS) levels and regions in Saudi Arabia. We hypothesized that the GMFCS level would affect service use for children with CP in Saudi Arabia. However, for the effect of region, we have no presumption.

## 2. Materials and Methods

### 2.1. Study Design and Ethical Consideration

This was a cross-sectional survey, based on parent interviews as well as child examination, conducted from February to August 2022. The study protocol followed ethical approval criteria according to the rules and regulations of the National Committee of Bioethics (NCBE) in Saudi Arabia with approval from the local Ethics Committee of the University of Tabuk (UT-175-39-2022).

### 2.2. Participants

A sample of convenience (*N* = 227) from 8 out of 13 provinces in Saudi Arabia (14 cities in total) of children with a diagnosis of CP and their parents were included in the study. For simplicity, we grouped the eight provinces into five geographic regions: north, south, central, east, and west (a common geographic grouping in Saudi Arabia).

Children were included in the study if they had a diagnosis of CP by their pediatric neurologist and met the criteria of the CP definition approved by the Surveillance of Cerebral Palsy in Europe [22]. However, the subtype classification of CP used for this study followed the Australian registers [23,24] describing tone abnormalities (e.g., spastic, dyskinetic, hypotonic, etc.) and topography (quadriplegia, diplegia, hemiplegia, and monoplegia).

### 2.3. Measures

#### 2.3.1. Child and Family Questionnaire

An electronic survey was developed by the research team (modified from a Jordanian study by Saleh and Almasri [16]) to gather demographic data on children and their families. Parents reported on their children’s associated health impairments from a list provided to them (including visual, hearing, speech, nutrition, learning, behavior, attention, epilepsy, respiratory, musculoskeletal, obesity, and pain problems). Parents were then asked to indicate whether treatments for the reported impairments were received (yes/no) and the extent to which each impairment affected the child’s activities of daily living (ADLs) on a scale of 1 (not at all) to 5 (to an extreme extent). Other information collected included past medical/surgical history and the school system the child attended. The questionnaire also collected information on the parents’ educational level, employment, total number of children in the family, presence of other children with a disability, language used at home, and overall family income.

#### 2.3.2. The Gross Motor Function Classification System-Expanded and Revised

The Gross Motor Function Classification System—expanded and revised [25,26,27] is a valid and reliable classification of child’s self-initiated gross motor functions, such as sitting, transfers, and mobility. Level I describes a child who walks without limitations, while level V describes a child who is transported in a manual wheelchair. Interrater reliability (kappa) was 0.55 for children less than 2 years of age and 0.75 for children 2 to 12 years of age [25,26,27]. Test–retest reliability was high (G = 0.79). The positive predictive value of the GMFCS at 1 to 2 years of age to predict walking by age 12 years was 0.74. The negative predictive value was 0.90 [25,26]. In this paper, we refer to this classification as GMFCS. The GMFCS levels for the participating children were determined by well-trained, criterion-tested pediatric physical therapists.

#### 2.3.3. Services Questionnaire

The electronic survey included questions on the use of specific medical and rehabilitation services, including family medicine, pediatrics, neurology, psychiatry, orthopedics, PT, occupational therapy (OT), speech therapy, and orthotics. Parents were asked if they had used these services for their children in the past 12 months (yes/no). If yes, parents were to report further on the frequency (sessions per year), intensity (the length of each session in minutes), and coverage of these services (free, private insurance, or payment). For PT, we also asked about the date of the first PT referral made and date of the first PT session taken (to calculate the waiting time for PT service). The whole questionnaire (child and family as well as service questionnaires) was discussed by a committee of three researchers (Saleh, M.N.; Alharbi, A.; Albalwi, A.) and research physical therapist (Alshahrani, A) to examine its clarity, comprehensiveness, and face validity. When finalized, it was pilot tested on 3 families of children with CP. Parents’ and research assistants’ feedback was then discussed among researchers and no major modifications were needed.

### 2.4. Procedure

Twelve research assistants who were physical therapists completed a two-hour training session conducted to explain the study procedures and measures before commencing with data collection. A manual explaining the survey questions and measures was prepared and provided to the RAs as a source of reference during the process of data collection. The participating families were recruited from public hospitals, except for the eastern region, where recruitment was from a private center. Research assistants contacted parents through their child’s physical therapists when they visited the hospital for their regular PT appointment. The aims, procedure, and expected outcomes of the study were explained and, if they agreed to participate, parents were provided with written informed consent forms. Parents (or a proxy) were interviewed by the research assistant to complete the survey and then the research assistant proceeded to assess the child to determine the GMFCS level and CP subtypes based on clear descriptions provided to them in the training session.

### 2.5. Statistical Analyses

The data were analyzed using IBM SPSS version 25 (SPSS Inc., Chicago, IL, USA). Descriptive analyses, including frequency tables for the demographics and children’s associated health problems, as well as treatment received for these impairments, were carried out. The total number of impairments each child had was then computed.

For medical and rehabilitation services, the total number of used services for each child (as reported by parents) in the past 12 months was calculated, as well as the mean service frequency and intensity. Moreover, a profile of the participating children (type of CP and associated health problems) and services provided to them were described by GMFCS levels. Inferential statistics (ANOVA for comparison of means and Chi-square for categorical data) were used to examine the associations of a child’s GMFCS level with impairments and with services (total number of services received). Finally, a second profile of children and services by regions in Saudi Arabia was conducted to fully describe and compare the characteristics of children and services by region. Inferential statistics were also constructed to examine the associations between the region and child’s associated impairments and services characteristics. A *p*-value of <0.05 was considered statistically significant a priori.

## 3. Results

### 3.1. Child and Family Characteristics

A sample of convenience (*N* = 227) of children (age range 6 months–18.2 years, mean 6.3 [SD 3.9] years) and their parents was included in the study. Child and family characteristics were available for all 227 children. Ninety-four children were born prematurely (41.4%). Table 1 shows the characteristics of participating children. Adolescents were less represented and there were more males than females.

Table 2 shows the characteristics of the participating families. More than two-thirds of families had a total of 2–4 children. In addition, 148 (65.2%) of the families had a monthly income of between 5000 and 15,000 Saudi Riyals (average income). As for parents’ characteristics, 172 (75.7%) of the mothers were under 41 years old, while 179 (78.8%) of the fathers were between 31 and 50 years old.

### 3.2. Children’s Associated Impairments

Thirty-four children (15%) were reported to have no associated impairments. For the rest of the children, the total number of impairments ranged from one problem (*n* = 38, 16.7%) to nine problems (*n* = 3, 1.3%). In addition, almost half of the children (*n* = 113, 49.8%) had three or more associated impairments. Table 3 shows associated impairments affecting children, the percentage receiving treatments for each impairment affecting them, and the extent each impairment affecting ADL. Learning and behavior problems were the highest to affect ADLs; however, less than one third of the children received treatment for these problems.

### 3.3. Previous Medical and Surgical Treatments

As for previous medical and surgical treatments, 61 (26.9%) of the children had had Botulinum Toxin A injections, 47 (20.7%) had had tendon release surgery, 16 (7%) had had serial casting, 7 (3.1%) had had other orthopedic surgeries, 3 (1.3%) had had neurologic surgery (such as dorsal rhizotomy or shunt), and only one child had a baclofen pump. In addition, 15 children (6.6%) had eye surgery and 36 (15.9%) were prescribed an Ankle Foot Orthoses (AFO).

### 3.4. School and Education

Services information was complete for 223 of children. Regarding school and education, 139 (62.3%) participating parents reported that their children did not attend school, and only one child was reported to attend an early intervention program. Additionally, 11 (4.9%) children were enrolled in kindergarten (governmental or private), 12 (5.4%) attended schools for children with special needs, and 38 (17%) were enrolled in ordinary governmental or private school settings. The average frequency of special education sessions per year was the highest among all services received (mean 71.75 [SD 70.8], range 10–285 sessions/year). This may be because some children received the service at the school they were attending, and this gave them the opportunity to access the service almost daily in the academic year.

### 3.5. Medical and Rehabilitation Services

For medical and rehabilitation services, 26 (11.7%) of the children had received only one medical/rehabilitation service in the past 12 months, and 7 (3.1%) received the highest number of services (6 services) (Table 4). Most of the children (*n* = 186, 83.4%) used between 2–5 types of medical and rehabilitation services. Specifically, 215 (96.4%) of the children in the study received PT, while 135 (60.5%) visited a pediatric neurologist, and 88 (39.5%) a pediatric orthopedist. On the other hand, 147 (65.9%) of the children did not receive OT services, and only 32 (14.3%) received speech therapy. Moreover, 102 (45.7%) had orthotic services.

Of the 215 children who received PT, 72 (33.5%) received the services once a week, 53 (24.7%) twice, and 32 (14.9%) three times a week. OT services which were received by only 76 (34.1%) children, were mainly once a week (*n* = 27, 35.5%) or once a month (*n* = 30, 39.5%). As for speech therapy, 20 of the 32 (62.5%) children who received the service received it once per month.

The average length of service session ranged from 29.1 min for neurologist visits (mean 29.1 [SD 23.6], *n* = 133) to 74 min for special education sessions (mean 74.2 [SD] 75.1, *n* = 24). Furthermore, the average waiting time for the first PT treatment (time difference between referral to PT and the actual start of sessions) was 1.7 months (SD = 6.6, Range 0–60, *n* = 212). Most of the participating children (*n* = 167, 77.8%) received their first PT session at the time of the referral without waiting.

Services were free for most of the children; for example, 67 of the 88 children (76%) who received orthopedist services received them free of charge. Free services were also received by 117 (86.7%) of the 135 children receiving neurologist services, 174 (80.9%) of the 215 children receiving PT, 68 (89.5%) of the 76 children receiving OT, 29 (90.6%) of the 32 receiving speech therapy, and 74 (72.5%) of the 102 receiving orthotic services. Those who did not receive free services either paid or had private insurance.

### 3.6. Profiles of Children and Services by GMFCS and by Region

There was a significant effect of the GMFCS levels on the total number of associated impairments a child had, *F* (4, 218) = 8.87, *p* < 0.001. Children in level I had a mean total of 1.5 (SD 1.86) associated impairments, while the mean value for children in level V was 4.0 (SD 2.1). However, the type and number of services did not differ as much as per the GMFCS levels. Table 5 shows the profiles of children and services as per GMFCS levels. PT, neurologist, and orthotic services were the most commonly used services for all GMFCS levels. Only neurologist services were significantly associated with GMFCS [*X*^2^ (4, *N* = 223) = 11.356, *p* = 0.022)], with more children in level I reported to have not received neurologist services (z = 2.0, *p* < 0.05).

A complete profile of the children’s characteristics and services by the geographic regions in Saudi Arabia is shown in Table 6. The children did not significantly differ in some characteristics (sex, age, and GMFCS) as per region. However, Fisher’s Exact test showed significant associations between region and impairments of learning (*p* = 0.046), behavior (*p* = 0.027), and attention (*p* = 0.023), as well as pain (*p* = 0.033).

Many of the medical services received significantly differed from one region to another as per Fisher’s Exact test. These included neurologist (*p* = 0.007), orthotics (*p* = 0.011), PT (*p* = 0.003), OT (*p* = 0.046), and speech therapy (*p* = 0.024). None of the children in the Eastern region were reported to have received speech therapy. In the same Eastern region, more children were reported to have not received neurologist services (z = 2.1, *p* < 0.05) and PT (z = 4.0, *p* < 0.001). Finally, the total number of medical services received differed significantly by region *F* (4, 218) = 3.47, *p* = 0.009. The northern region reported the highest mean of 3.6 (SD 1.3, *n* = 7), while the eastern region reported the lowest (mean 2.3, SD 1.4, *n* = 28).

## 4. Discussion

In this study, we aimed to explore the characteristics of children with CP and service utilization in Saudi Arabia. Similarly, we sought to examine how services differed according to GMFCS levels and regions in Saudi Arabia. To our knowledge, this was the first study to explore characteristics of children with CP and service utilization in Saudi Arabia. Most children were of the more severe types of CP (spastic quadriplegia and diplegia). In addition, almost half of our sample were walkers with different degrees of limitations (GMFCS levels I–III), and the rest were in GMFCS levels IV and V (more severely limited), which indicates a fair distribution among motor functional levels. Furthermore, although more than 75% of children were above three years old, our sample included only 17 adolescents (>12–18 years); therefore, our results may not apply to adolescents with CP. The low number of participating adolescents may be explained by the low number of adolescents attending health centers where the sample was obtained. The literature has shown that as children with disabilities grow up, they receive less health care [16,28,29,30]. It is worth noting that the families were of average income, with well-educated, young–middle-aged parents, reflecting the normal population of Saudi Arabia [19]. PT was the most-used service in the previous 12 months for almost all children. Our initial hypothesis that service utilization would differ based on the GMFCS levels was not verified.

### 4.1. Children with CP and Their Families in Saudi Arabia

Children’s characteristics in this study were comparable with those of other previous studies. For example, there were more boys than girls, which was reported in other studies [16,31,32,33,34,35,36]. Furthermore, spastic CP was the predominant type of CP in the aforementioned studies, and in the present study. Similar to our study, studies from developing countries included young children (less than 5 years) comprising one third or more of their samples [13,35,37]. Although many registries consider 5 years of age to be the optimal age for the confirmation of the diagnosis of CP [22], new scientific evidence now exists which supports early diagnoses [38,39,40]). Undoubtedly, early diagnosis will ensure that the affected child receives CP-specific early intervention. Therefore, although the diagnoses for some of our young children may not be final, they were already reported to have received management for CP, which is an advantage. That being said, we recommend follow-up examinations every 6/12 months to confirm the diagnosis and the GMFCS level for these children [41].

It is widely known now that although CP is defined as a problem of posture and movement, affected children experience a variety of associated impairments that are not motor. In the present study, half of the children had three or more associated impairments other than motor disorders, with speech and visual problems being the most frequent impairments. Visual problems received more treatments than speech problems and were less likely to affect children’s ADL. Other impairments which affected children’s daily activities and received less treatment were behavior, learning, and attention disorders. We understand that parents may have overestimated the effect of untreated impairments on a child’s daily activities; however, clinicians usually pay more attention to motor problems for these children and overlook other problems, such as behavior, attention, and speech problems [42]. Failing to address these associated impairments places an additional burden on the families. The recent literature is now paying attention not only to motor impairments of CP, but also to associated impairments [5,35,37,43]. Since OT, speech therapists’, and special educators’ services were reported to have been used less in our study, we recommend a change of practice. A multidisciplinary approach to the management of CP will help address all associated problems which are now considered part of the clinical picture of CP [4]. It is worth noting as well that speech, behavior, learning, and attention disorders may have affected the children’s integration at school. This may explain, at least in part, the decreased access of these children to the school system. The significant associations between region and impairments of learning, behavior, attention, and pain are worth more investigations to explore reasons for these differences. Since we used parents’ reporting, the differences could be related to differences in parents’ awareness of the problem and how to detect it among different regions. The age of the child (which differed slightly by region) may also have affected the detection of these problems by parents.

Almost all participating families have only one child with a disability. Parents and family characteristics did not differ from the normal population of Saudi Arabia. The mothers of the children in the study were not working (which is a common situation in Saudi Arabia) and were young (63.2% of the population in Saudi Arabia are younger than 35 years old [19]) and well-educated, and the family received an average income, and had a small number of children (the average household size in Saudi Arabia is 4.8) [19]. Based on this, it is expected that the affected children would have more attention and support from their families, particularly given that most of them live with both parents. However, it is worth studying the effects of family characteristics on a child’s functional limitations and service utilization.

### 4.2. Services for Children with CP in Saudi Arabia

Most of the participating children had spasticity. However, medical and surgical treatments for spasticity (e.g., baclofen, Botulinum Toxin A, dorsal rhizotomy, etc.) were not used very often. Nonetheless, Botulinum Toxin A treatment was the spasticity treatment most often used. The effectiveness of Botulinum Toxin A in the management of CP is still not conclusive [44,45,46]. Therefore, we recommend assessing the short- and long-term effects of its use for children in Saudi Arabia before adopting it as a regular treatment.

The number of children attending school (including preschool and kindergarten) was considerably low. More than half of our sample were under 6 years old, and this may, in part, explain the results. However, since more than 75% of them were above three years old and half of our sample were of GMFCS levels I–III (walkers with different degrees of limitations), we think that these children should be enrolled in the school system (including early intervention programs). Reasons for not being in schools could be related to environmental barriers, child factors, or family resources. Colver et al. [47] reported that children suffering from CP with impaired walking ability had less access to the physical environment and transport when compared to other children. In our study, associated problems, which may relate to education and school, appeared to be common among children of all GMFCS levels. These include visual, learning, attention, and speech problems. Many parents reported that their children did not receive treatment for these problems, which may have contributed to decreased access to school and other educational services. Further research is needed for a deeper exploration of why children with CP did not attend schools. The findings can contribute to making the Saudi educational programs and systems more accessible to children with disabilities.

PT services were the most-used service across GMFCS levels and across regions. The mean waiting time for the first PT session was almost the same as the mean reported by a Jordanian study conducted by Saleh and Almasri [16], which was 1.02 [SD 2.9] months. However, it is lower than the waiting times reported in by Feldman et al. [9], with a mean of 129.4 [SD 51.6] days, and by Grilli et al. [10], with a mean of 6.6 [SD 5.1] months. The short or lack of waiting time for children to receive their first PT session in Saudi Arabia is an interesting finding and is expected to have a positive impact on outcomes for these children. Further research is needed to address factors leading to decreased waiting time in Saudi Arabia compared to other parts of the world [9,10].

As expected, children with different GMFCS levels reported different impairments. The common associated impairments for all levels of GMFCS included speech, visual, attention, and learning problems. However, certain problems, such as feeding and orthopedic problems, as well as epilepsy disorders, appeared to be more ubiquitous in the levels deemed to have greater severity (IV and V). It is worth noting that regardless of the type of impairment, PT, neurologist, and orthotic services were the highest services used by most of the children across GMFCS levels. These findings may indicate that medical and rehabilitation services were accessible to all children regardless of their functional limitations, which are positive and encouraging findings. However, some services were not sufficiently used as needed, such as OT and speech therapy. The reasons for the decreased utilization of these services by children with CP need to be further explored. In addition, more research is needed on how to make these services more accessible to these children.

In general, health services were provided free of charge most of the time and were available in almost all regions (with a few exceptions, such as occupational therapy and speech therapy). Our findings prove the health system’s success in providing these services across the entire country. Some differences in service use in the northern region may be explained by the small sample size in this region. Additionally, children from the eastern region were all recruited from private settings, which may explain their decreased use of many services.

There are some limitations that need to be considered when trying to interpret our results. Our sample included children who are under 5 years old, as we took a sample of convenience of children attending hospitals. Therefore, the results may not apply to adolescents (12–18 years). In addition, we used parent report in the present study to collect demographics and services data, while CP classification and type were assessed by experienced physical therapists. Parent reporting is used widely in CP research and registers [13,24]; however, we understand that it may be affected by recall bias or overestimation. Finally, this study covered many provinces in Saudi Arabia that represent the five geographic regions. The largest region by population size is the central region (which includes the capital city), followed by the western region, then the Eastern [19]. We aimed to recruit a number of participants from each region that is proportionate to its population size. However, the east and north regions were less represented because we were not able to recruit enough research assistants in these regions. Thus, the results may not fully apply to the aforementioned regions. Nevertheless, the study sample was adequate to give a clear vision of the characteristics of children in Saudi Arabia and their use of services.

### 4.3. Implication for Practice

Management for children with CP needs to be comprehensive and multidisciplinary. Addressing non-motor impairments is essential to reduce its effect on ADLs and school integration. Similarly, planning individualized management for each child based on his/her motor and non-motor impairments may support the provision of quality and efficient services.

## 5. Conclusions

This study was the first to explore the characteristics of children with CP in Saudi Arabia and the services delivered. Our children’s characteristics and associated impairments did not differ by GMFCS. However, there were many gaps in service delivery that need to be addressed. Associated impairments for children with CP need to be addressed, along with motor problems, with adequate facilitation of the use of OT, speech therapy, and educational services as needed. Data on service use and on unmet needs for children may support the proper and efficient allocation of services to maximize benefits to children and provide more comprehensive rehabilitation services.

## Figures and Tables

**Table 1:** Children’s characteristics (*N* = 227).

Children Characteristics	*n* (%)
Children’s age groups (years)	
Infants and toddlers (0–3)	56 (24.7)
Preschoolers (>3–6)	71 (31.3)
School-age (>6–12)	83 (36.6)
Adolescents (>12–18)	17 (7.5)
Child sex	
Male	134 (59.0)
Female	93 (41.0)
GMFCS levels	
Level I	21 (9.3)
Level II	56 (24.7)
Level III	46 (20.3)
Level IV	52 (22.9)
Level V	52 (22.9)
Predominant motor types and subtypes of cerebral palsy	
Spastic	182 (80.2)
Quadriplegia	66 (29.1)
Diplegia	78 (34.4)
Triplegia	11 (4.8)
Hemiplegia	22 (9.7)
Monoplegia	5 (2.2)
Mixed	7 (3.1)
Hypotonia	19 (8.4)
Athetoid	6 (2.6)
Ataxic	5 (2.2)
Unknown	8 (3.5)

GMFCS: Gross Motor Function Classification System.

**Table 2:** Parents’ and family characteristics (*N* = 227).

Parents’ Characteristics	*n* (%)
Mothers’ age-group (years)	
21–30	72 (31.7)
31–40	100 (44.0)
41–50	49 (21.6)
≥51	6 (2.6)
Fathers’ age-group (years)	
21–30	19 (8.4)
31–40	108 (47.6)
41–50	71 (31.3)
≥51	29 (12.8)
Mothers’ Educational level	
Less than primary school	10 (4.41)
Primary school	18 (7.9)
Intermediate school	16 (7.0)
High school	82 (36.1)
Diploma	17 (7.5)
Bachelor	81 (35.68)
Masters	2 (0.88)
PhD	1 (0.4)
Fathers’ Educational level	
Less than primary school	5 (2.2)
Primary school	14 (6.2)
Intermediate school	17 (7.5)
High school	83 (36.6)
Diploma	31 (13.7)
Bachelor	71 (31.3)
Masters	5 (2.2)
PhD	1 (0.4)
Mothers’ employment status	
Full-time	35 (16.1)
Part-time	5 (2.3)
Not working	187 (81.6)
Fathers’ employment status	
Full-time	174 (76.6)
Part-time	9 (4.0)
Not working	44 (19.4)
Total number of children in family *	
1	48 (21.1)
2	63 (27. 8)
3	46 (20.3)
4	44 (19.4)
5	18 (7.9)
≥6	8 (3.5)
Other children with disabilities in family	
Yes	33 (14.5)
No	194 (85.5)
Family income/month **	
≥15,000	37 (16.3)
10,001–15,000	51 (22.5)
5000–10,000	97 (42.7)
<5000	42 (18.5)
Relationship of respondent to the child	
Mother	182 (80.2)
Father	37 (16.3)
Both parents	2 (0.8)
Others	6 (2.6)

* Including the participant child, ** Saudi Riyal.

**Table 3:** Associated impairments and treatments received (*N* = 227).

Impairment	Affected Children *n* (%)	Received Treatment *n* (%)	ADL ^†^ Mean (SD)
Speech impairment	124 (54.6)	44 (35.5)	2.21 (1.4)
Visual impairment	87 (38.3)	69 (79.3)	1.38 (1.4)
Learning problems	85 (37.4)	28 (32.9)	2.51 (1.3)
Attention problem	68 (30.0)	23 (33.8)	2.24 (1.2)
Feeding problem	56 (24.7)	36 (64.3)	2.23 (1.3)
Epilepsy disorders	50 (22.0)	47 (94.0)	1.68 (1.3)
Orthopedic conditions	49 (21.6)	35 (71.4)	2.08 (1.4)
Behavior problems	35 (15.4)	8 (22.9)	2.34 (1.4)
Pain	34 (15.0)	21 (61.8)	2.03 (1.3)
Respiratory disease	29 (12.8)	21 (72.4)	2.03 (1.5)
Hearing impairment	13 (5.70)	9 (69.2)	2.15 (1.6)
Obesity	5 (2.20)	0 (00.0)	2.20 (0.8)

ADL: activities of daily living. **^†^** Impairment affected ADL on a scale (1: not at all–5: extreme extent).

**Table 4:** Total number of services a child received * (*N* = 223).

Number of Services	*n* (%)
0	4 (1.8)
1	26 (11.7)
2–3	124 (55.6)
4–5	62 (27.8)
6	7 (3.1)

* As per parent report.

**Table 5:** Characteristics of Children and Services by GMFCS (*N* = 223).

GMFCS Level	I (*n* = 21)	II (*n* = 56)	III (*n* = 46)	IV (*n* = 50)	V (*n* = 50)
Age, years Mean (SD)	6.8 (4.1)	6.7 (3.5)	5.1 (2.8)	5.8 (4.3)	6.6 (3.8)
Distribution of CP *	*n* (%)	*n* (%)	*n* (%)	*n* (%)	*n* (%)
Quadriplegia	-	5 (8.9)	3(6.5)	19 (38.0)	37 (74.0)
Triplegia	-	-	4 (8.7)	-	2 (4.0)
Diplegia	8 (38.1)	23 (41.1)	29 (63.0)	14 (28.0)	2 (4.0)
Hemiplegia	5 (23.8)	14 (25.0)	-	-	-
Hypotonia	4 (19.0)	-	3 (6.5)	6 (12.0)	4 (8.0)
Dyskinetic	-	-	-	-	2 (4.0)
Associated impairments **					
Speech	5 (23.8)	24 (42.9)	19 (41.3)	28 (56.0)	44 (88.0)
Learning	4 (19.0)	18 (32.1)	14 (30.4)	14 (28.0)	31 (62.0)
Attention	4 (19.0)	14 (25.0)	14 (30.4)	14 (28.0)	18 (36.0
Visual	4 (19.0)	16 (28.6)	20 (43.5)	25 (50.0)	22 (44.0)
Orthopedic	4 (19.0)	-	-	13 (26.0)	14 (28.0)
Feeding	-	-	-	14 (28.0)	24 (48.0)
Epilepsy	-	-	-	-	20 (40.0)
Services received ***					
PT	19 (90.5)	54 (96.4)	44 (95.7)	50 (100.0)	48 (96.0)
Orthopedist	12 (57.1)	19 (33.9)	22 (47.8)	22 (44.0)	13 (26.0)
Neurologist	7 (33.3)	31 (55.4)	30 (65.2)	30 (60.0)	37 (74.0)
Orthotics	11 (52.4)	26 (46.4)	22 (47.8)	21 (42.0)	22 (44.0)
OT	-	18 (32.1)	17 (37.0)	19 (38.0)	19 (38.0)

* (displays the most common 3 types); ** (displays those with ≥20% except for level I); *** (displays those with ≥25%); GMFCS: Gross Motor Function Classification System; CP: cerebral palsy; PT: physical therapy; OT: occupational therapy.

**Table 6:** Profile of children and services by region (*N* = 223).

Region		South (*N* = 51)	Center (*N* = 49)	East (*N* = 28)	West (*N* = 88)	North (*N* = 7)
GMFCS levels *n* (%)	I	4 (7.8)	6 (12.2)	6 (21.4)	5 (5.7)	0 (0)
	II	16 (31.4)	11 (22.4)	7 (25.0)	21 (23.9)	1 (14.3)
	III	10 (19.6)	12 (24.5)	2 (7.1)	20 (22.7)	2 (28.6)
	IV	12 (23.5)	11 (22.4)	7 (25.0)	18 (20.5)	2 (28.6)
	V	9 (17.6)	9 (18.4)	6 (21.4)	24 (27.3)	2 (28.6)
Age in years mean (SD)		5.97 (3.7)	5.31 (3.3)	7.54 (5.0)	6.27 (3.4)	7.05 (4.4)
Sex *n* (%)	Male	30 (58.8)	29 (59.2)	17 (60.7)	51 (58.0)	4 (57.1)
	Female	21 (41.2)	20 (40.8)	11 (39.3)	37 (42.0)	3 (42.9)
Associated impairments *^†^ *n* (%)	Speech	29 (56.9)	20 (40.8)	13 (46.4)	53 (60.2)	5 (71.4)
	Visual	16 (31.4)	23 (46.9)	12 (42.9)	31 (35.2)	5 (71.4)
	Attention			**14 (50.0)**	**28 (31.8)**	
	Learning	**25 (49.0)**		**11 (39.3)**	**32 (36.4)**	**3 (42.9)**
	Feeding		17 (34.7)			4 (57.1)
	Pain		**13 (26.5)**	**7 (25.0)**		
	Orthopedic			10 (35.7)	24 (27.3)	
	Epilepsy					3 (42.9)
	Behavior			**7 (25.0)**		**2 (28.6)**
	Respiratory					2 (28.6)
Services received *^†^ *n* (%)	PT	**51 (100.0)**	**49 (100.0)**	**23 (82.1)**	**85 (96.6)**	**7 (100.0)**
	Neurologist	**31 (60.8)**	**37 (75.5)**	**10 (35.7)**	**51 (58.0)**	**6 (85.7)**
	Orthotics	**29 (56.9)**	**25 (51.0)**	**17 (60.7)**	**28 (31.8)**	**3 (42.9)**
	Orthopedist	**15 (29.4)**	**24 (49.0)**	**10 (35.7)**	**35 (39.8)**	**4 (57.1)**
	OT	**16 (31.4)**	**17 (34.7)**		**38 (43.2)**	
	Speech Therapy					**3 (42.9)**
Total number of services received ** *n* (%)	0	0 (0)	0 (0)	3 (10.7)	1 (1.1)	0 (0)
	1	8 (15.7)	1 (2.0)	7 (25.0)	10 (11.4)	0 (0)
	2–3	27 (52.9)	31 (63.3)	11 (39.3)	52 (59.1)	3 (42.9)
	4–6	16 (31.4)	17 (34.7)	**7 (25.0)**	25 (28.4)	**4 (57.1)**

* Displays those with >25%; **bold text indicates *p* < 0.5**; ^†^ Chi-square/Fisher’s Exact test; ** ANOVA; GMFCS: Gross Motor Function Classification System; PT: physical therapy; OT: occupational therapy.

## Data Availability

Restrictions apply to the datasets: The datasets presented in this article are not readily available for ethical and patient privacy considerations. Requests to access the datasets should be directed to the corresponding author (MS).
